# Thoracoscopic Esophagectomy for a Huge Leiomyosarcoma

**DOI:** 10.1055/s-0039-1696729

**Published:** 2019-10-22

**Authors:** John Mathew Manipadam, Satinder Pal Singh Bains, S. Mahesh, Ami Maria Emmanuel, H. Ramesh

**Affiliations:** 1 Department of GI Surgery and Liver Transplantation, VPS Lakeshore Hospital and Research Centre, Kochi, Kerala, India; 2 Department of Pathology, VPS Lakeshore Hospital and Research Centre, Kochi, Kerala, India

**Keywords:** leiomyosarcoma of esophagus, thoracoscopic esophagectomy, minimally invasive esophagectomy

## Abstract

Esophageal leiomyosarcoma is the commonest of all esophageal sarcomas but yet has a very low incidence. These tumors have been resected by the open approach so far. We describe the steps and challenges involved in the thoracoscopic excision of a huge leiomyosarcoma of the esophagus.

Leiomyosarcoma of the esophagus is a rare disease. 1 These tumors grow slowly and attain a massive size and are malignant in nature with surrounding infiltration. 2 We describe the steps and challenges involved in the thoracoscopic excision of a huge leiomyosarcoma of the esophagus. Thoracoscopic esophagectomy for a leiomyosarcoma of the esophagus has not been reported in literature so far.

## Preoperative Preparation

A 68-year-old male presented to us with dysphagia for solids, weight loss and right sided chest pain since 2 months. There were no comorbidities or any previous surgery. General and systemic examination did not reveal any abnormality except for a low body mass index (BMI) of 15.43 kg/m ^2^ .

**Fig. 1:**
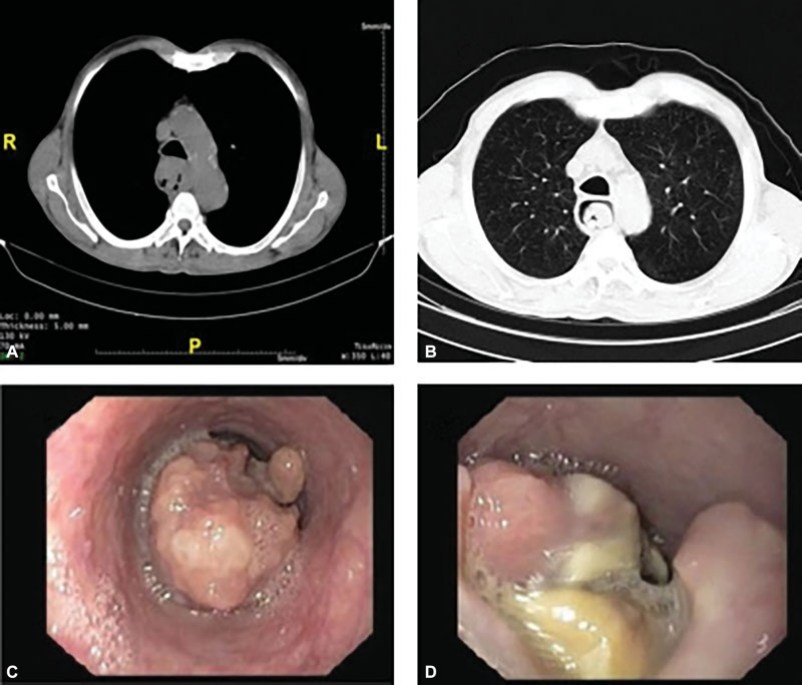
(A,B) Computed tomography images of the tumor. (C,D) Endoscopic images of tumor.

Contrast enhanced computed tomography scan of the chest revealed an enhancing circumferential mural thickening over a length of 8 to 10 cm of midthoracic esophagus with an intraluminal polypoidal component ( Fig. 1A ). Upper gastrointestinal endoscopy showed a large ulceroproliferative growth with overhanging edges extending from 22 to 38 cm from incisors involving more than three-fourths of the circumference ( Fig. 1B–D ). Biopsy was reported as poorly differentiated spindle cell neoplasm. Cardiopulmonary and anesthetic fitness was ascertained. We initiated Incentive spirometer for respiratory fitness and high-protein supplements in view of low BMI. Patient was counseled, informed consent was obtained, and scheduled for a thoracoscopic esophagectomy followed by gastric pull-up.

## Positioning of Patient and Ports

**Fig. 2:**
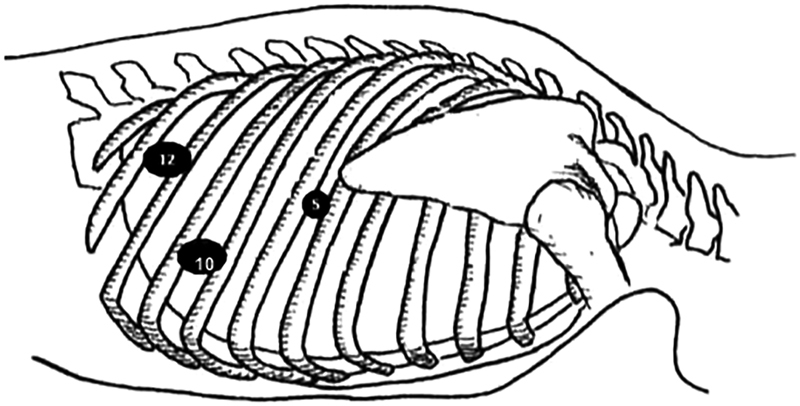
Port position for thoracoscopic esophagectomy.

Intubation was done with a single-lumen endotracheal tube in the supine position after which the patient was placed in prone position and the right side of the chest was painted and draped. The ports placed were a 10-mm port in the 10th intercostal space 15 cm lateral to the spine for the left working hand, 10-mm port in the 8th intercostal space in the mid axillary line for the camera, and a 5-mm port in the 6th intercostal space adjacent to the angle of the scapula for the right working hand. The 10th intercostal space port was later changed to 12 mm for the insertion of vascular stapler ( Fig. 2 ). The surgeon and the assistant stood on the patient's right side with the monitor on his left. Pneumomediastinum was created using carbon dioxide insufflation up to a pressure of 6 to 8 mm Hg.

## Operative Steps

**Fig. 3:**
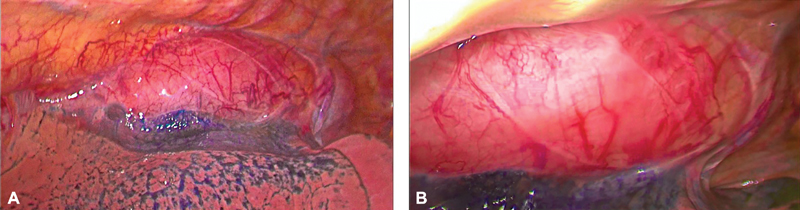
(A,B) Initial thoracoscopic view of huge tumor stretching the azygos vein.

Intraoperatively, we found a large esophageal tumor occupying the upper and midthoracic esophagus, predominantly intraluminal, pushing and stretching the adjacent structures, especially the azygos vein at its entry to the superior vena cava (SVC) ( Fig. 3A , B )

**Fig. 4:**
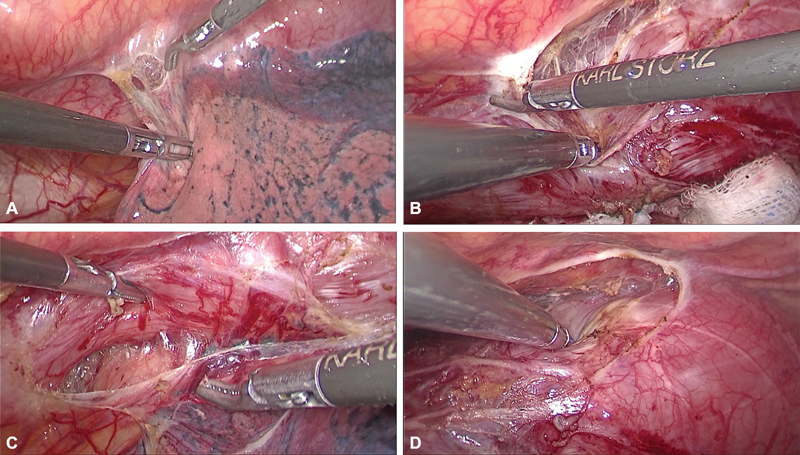
(A) Division of inferior pulmonary ligament. (B) Incising mediastinal pleura posterior to esophagus. (C) Incising mediastinal pleura anterior to esophagus. (D) Mobilising the tumor.

**Fig. 5:**
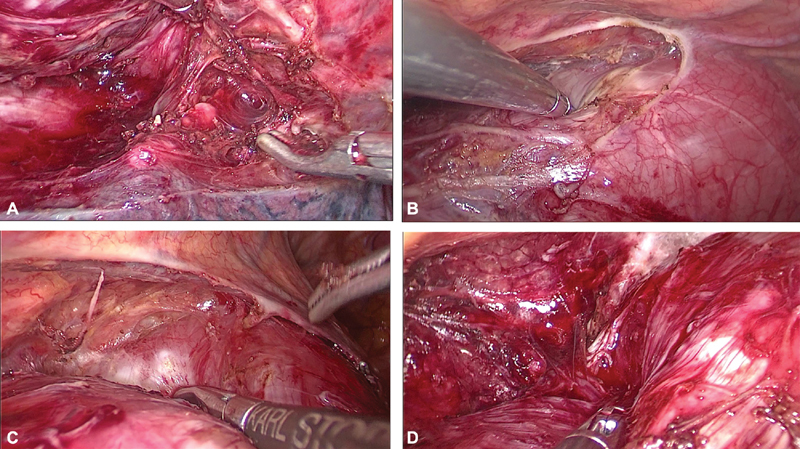
(A) Separating from the carina. (B) Separating from the aorta. (C) Dissecting away the azygos vein. (D) Abundant neovascularity in the adhesions around the tumor.

**Fig. 6:**
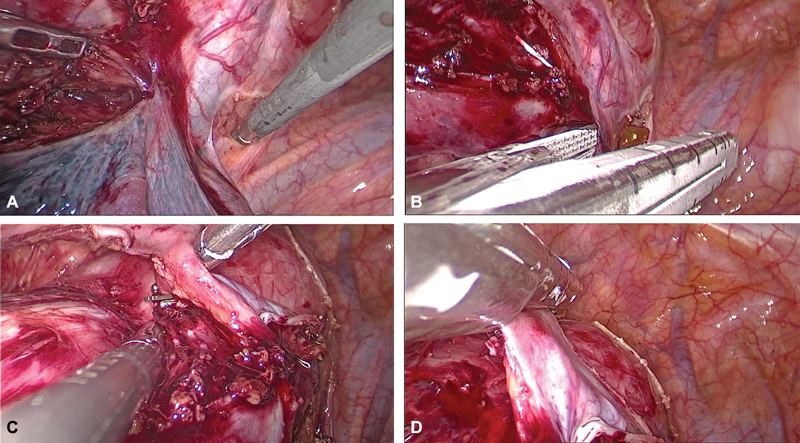
(A–D) Tackling the azygos vein.

**Fig. 7:**
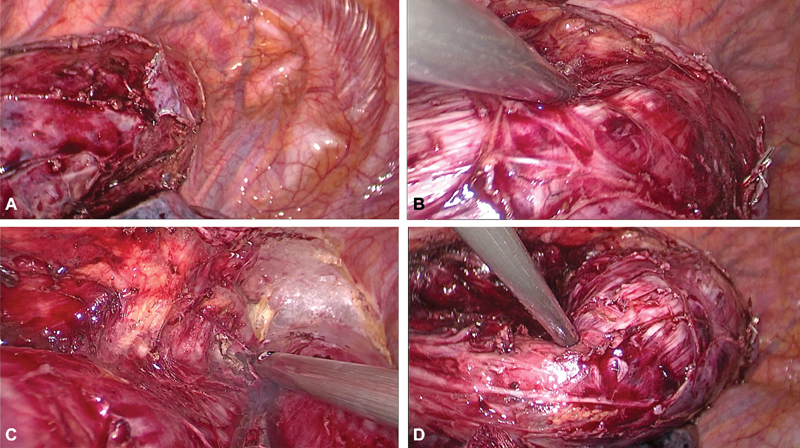
(A,B) Disconnecting the azygos helps in lateral mobilisation of the tumor. (C,D) Separating from the arch of the aorta.

**Fig. 8:**
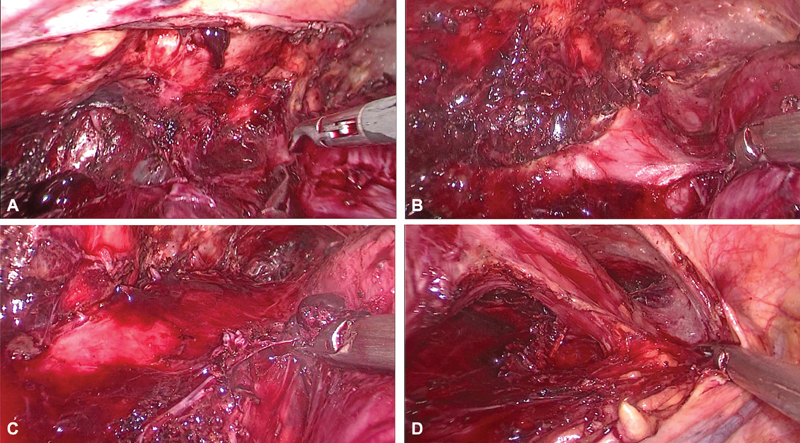
(A–C) Separating from the trachea. (D) Dissecting the normal esophagus above the tumor.

**Fig. 9:**
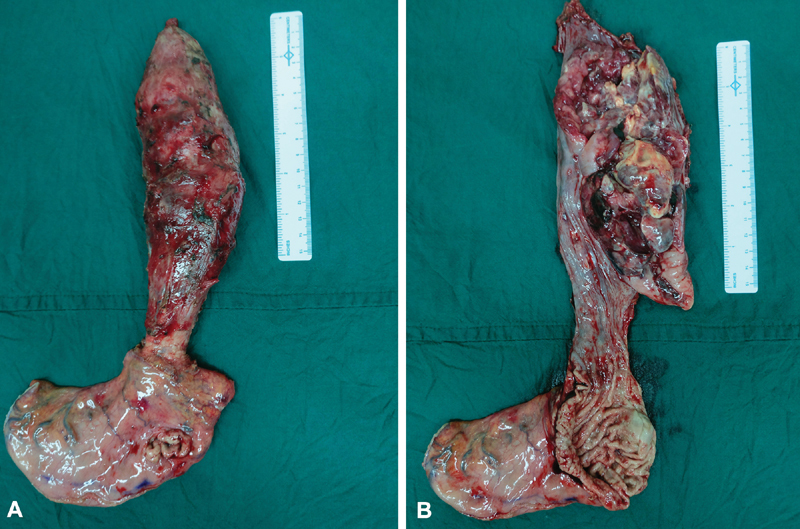
(A,B) Gross specimen.

Careful and meticulous dissection was performed. The inferior pulmonary ligament was divided ( Fig. 4A ), mediastinal pleura anterior and posterior to the thoracic esophagus were incised (commencing away from the tumor bearing esophagus; Fig. 4B , C ). Further mobilization of the tumor area was preceded with ( Fig. 4D , Video 1 ). Separating the growth from the right and left main bronchus, carina, arch of aorta, and the azygos vein was indeed a challenge, since the bulky mass was wedged in between these vital structures with considerable adhesions and obscuring of anatomy but no infiltration ( Fig. 5A–C ). There was considerable neovascularization in the adhesions to the surrounding structures ( Fig. 5D ). To create space to dissect around the tumor the azygos vein was sacrificed. It was stapled twice by a curved tip vascular stapler through the tenth intercostal port above close to the spine and below adjacent to its entry into the superior vena cava ( Fig. 6A–D ; Video 2 ). The intervening segment of the azygos vein was removed along with the tumor ( Fig. 7A ). This enabled us to apply some traction on the esophageal mass to the right with the left hand working port to dissect the vascular adhesions to the arch of the aorta and the underlying tracheobronchial apparatus ( Figs. 7B–D , and 8A–C ; Video 3 ) At the thoracic inlet, we found that the esophagus appeared normal ( Fig. 8D ). The thoracoscopic procedure was completed by subcarinal and supracarinal lymph node dissection and a right-sided intercostal drain was placed after ensuring hemostasis and normal lung expansion. The patient was then placed in a supine position for a supraumbilical midline laparotomy and mobilization of the stomach (greater curve stomach tube) for a routine gastric pull-up and a subsequent cervical esophagogastric anastomosis. Cut section of the resected specimen showed an 18 × 9 cm ulceroproliferative growth occupying nearly three-fourths of the circumference ( Fig. 9A , B ). Blood loss was 50 mL and total operative time was 9 hours.

**Video 1**

Initial steps of thoracoscopic esophageal mobilization.

**Video 2**

Tackling the azygos vein.

**Video 3**

Further and final steps in esophageal mobilization.

Postoperative recovery was uneventful except for a minor chylous leak which settled with a brief period (5 days) of conservative treatment, medium chain triglyceride based diet and feeds through the feeding jejunostomy. Oral liquids were commenced from the postoperative day 5 after an oral gastrografin contrast examination. This was gradually progressed to a semisolid diet and patient was discharged on the postoperative day 14.

**Fig. 10:**
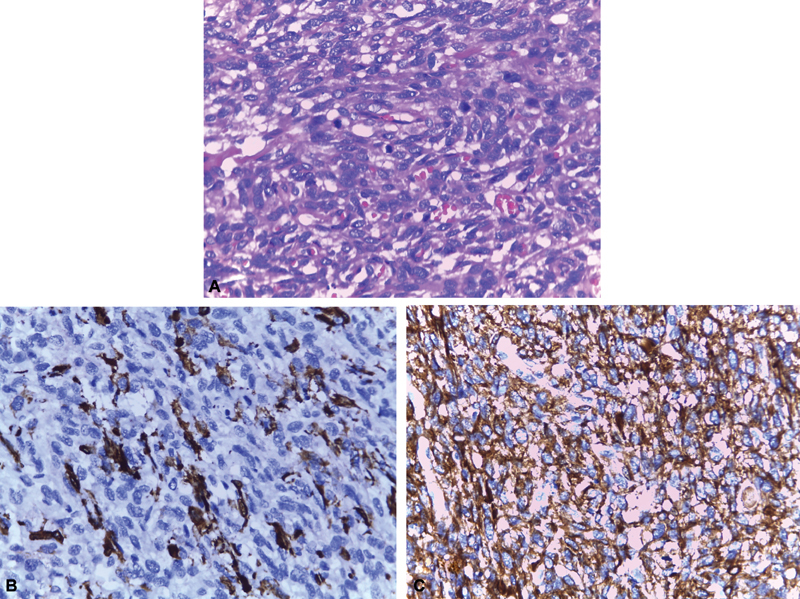
(A) Hematoxylin and Eosin stain. (B,C) Immunohistochemistry for smooth muscle actin and caldesmon.

Histopathology revealed a transmural infiltrating neoplasm composed of spindly to ovoid and polygonal cells with ovoid to irregular pleomorphic nuclei and coarse chromatin. Mitotic figures (35/10 high power field [hpf]), Necrosis (10%), myxoid change, hyalinization, multinucleated cells, and cells with bizarre nuclei were also present ( Fig. 10A ). The morphology and immunohistochemistry (IHC) (positive for smooth muscle actin and caldesmon) were suggestive of leiomyosarcoma; French Federation of Cancer Centers (FNCLCC) grade 3( Fig. 10B ). Tumor reached up to radial margins in some areas but the proximal and distal margins were free. There was no lymphovascular invasion. The periesophageal and subcarinal nodes were free of disease.

The patient recovered smoothly and is on normal diet. A multidisciplinary board has recommended postoperative radiotherapy.

## Discussion

Leiomyosarcoma of the esophagus is the most common of the esophageal sarcomas with only over 200 cases reported in the literature till today. 3 It accounts for 0.5% of all esophageal and 5% of all gastrointestinal sarcomas. 4 Only three case series have been reported in literature, the study period ranging from 29 to 76 years and the numbers in those time periods being 5 to 17. 2 3 4 This evidence points toward the rarity of the disease. An excellent prognosis with radical resection has been recorded for leiomyosarcoma esophagus in these series and even salvage surgical resection is recommended in patients with resectable metastases. 2 Several factors determine the success of this surgical exercise, such as the size of the tumor, its infiltration of the surrounding vital structures, local and distant metastases, comorbidities of the patient, and finally the performance status of the patient to withstand the stress of such a major surgical undertaking.

To the best of our knowledge, thoracoscopic mobilization for leiomyosarcoma of the esophagus has not been described in literature. Apart from a mention of two minimally invasive approaches without details in a case series of esophageal sarcomas in general 3 and isolated endoscopic and local resections for small polypoidal variants, 4 5 the vast majority of esophageal leiomyosarcomas have been resected through the open approach. 6 7 8 9 10 The reason probably is the fact that these tumors have been known to attain sizes as large as 16 cm in length and present as mediastinal masses with deep ulcerations. 2 However, Zhang et al noted in his case series that most of these tumors showed expansive growth and less infiltration of surrounding organs, and hence the dissection was relatively easy in most cases. 2 This justifies the thoracoscopic approach for our patient who had a tumor length of nearly 17 cm and a width of 8 cm. The patient had a smooth postoperative recovery with little pain and respiratory complications compared with an open transthoracic approach. In the largest ever review of smooth muscle tumors of esophagus, Hatch et al reported that mortality associated with surgery for leiomyosarcoma of the esophagus is high in the immediate postoperative period due to pulmonary and anastomotic complications. 8 This may serve to emphasize the importance of approaching esophageal leiomyosarcomas by minimally invasive approach in surgery even when there is larger tumor size. The new knowledge that we gain from this report that can be applied in routine clinical practice is that esophageal leiomyosarcomas, though huge in size, can indeed be removed thoracoscopically with resultant significant decrease in postoperative morbidity and an uneventful recovery after operation.
